# *Waddlia chondrophila* Infects and Multiplies in Ovine Trophoblast Cells Stimulating an Inflammatory Immune Response

**DOI:** 10.1371/journal.pone.0102386

**Published:** 2014-07-10

**Authors:** Nick Wheelhouse, Christopher Coyle, Peter G. Barlow, Stephen Mitchell, Gilbert Greub, Tim Baszler, Mick T. Rae, David Longbottom

**Affiliations:** 1 Moredun Research Institute, Edinburgh, Midlothian, United Kingdom; 2 School of Life, Sport and Social Sciences, Edinburgh Napier University, Sighthill Campus, Edinburgh, United Kingdom; 3 Institute of Molecular Plant Sciences, University of Edinburgh, Edinburgh, United Kingdom; 4 Center for Research on Intracellular Bacteria (CRIB), Institute of Microbiology, University Hospital Center and University of Lausanne, Lausanne, Switzerland; 5 Washington Animal Disease Diagnostic Laboratory, College of Veterinary Medicine, Washington State University, Pullman, Washington, United States of America; Auburn University, United States of America

## Abstract

**Background:**

*Waddlia chondrophila* (*W. chondrophila*) is an emerging abortifacient organism which has been identified in the placentae of humans and cattle. The organism is a member of the order Chlamydiales, and shares many similarities at the genome level and in growth studies with other well-characterised zoonotic chlamydial abortifacients, such as *Chlamydia abortus* (*C. abortus*). This study investigates the growth of the organism and its effects upon pro-inflammatory cytokine expression in a ruminant placental cell line which we have previously utilised in a model of *C. abortus* pathogenicity.

**Methodology/Principal Findings:**

Using qPCR, fluorescent immunocytochemistry and electron microscopy, we characterised the infection and growth of *W. chondrophila* within the ovine trophoblast AH-1 cell line. Inclusions were visible from 6 h post-infection (p.i.) and exponential growth of the organism could be observed over a 60 h time-course, with significant levels of host cell lysis being observed only after 36 h p.i. Expression of CXCL8, TNF-α, IL-1α and IL-1β were determined 24 h p.i. A statistically significant response in the expression of CXCL8, TNF-α and IL-1β could be observed following active infection with *W. chondrophila*. However a significant increase in IL-1β expression was also observed following the exposure of cells to UV-killed organisms, indicating the stimulation of multiple innate recognition pathways.

**Conclusions/Significance:**

*W. chondrophila* infects and grows in the ruminant trophoblast AH-1 cell line exhibiting a complete chlamydial replicative cycle. Infection of the trophoblasts resulted in the expression of pro-inflammatory cytokines in a dose-dependent manner similar to that observed with *C. abortus* in previous studies, suggesting similarities in the pathogenesis of infection between the two organisms.

## Introduction

*Waddlia chondrophila* is an emerging pathogen belonging to the order Chlamydiales. The Chlamydiales are Gram-negative obligate intracellular pathogens that cause a range of pathogenic conditions in a wide variety of host species [1]. All known members of the order share a similar, distinct biphasic developmental cycle, initiated by entry of the infectious form of the organism, the elementary body (EB), into the host cell where it resides within a vacuole known as an inclusion. The EB undergoes conversion to the metabolically active reticulate body (RB), which replicates through binary fission. Towards the end of the cycle the RBs re-condense to EBs prior to lysis of both the inclusion and the host cell, allowing release of infective organisms to infect neighbouring cells [1].

*Waddlia chondrophila* was originally isolated in 1986 from a bovine fetus [2] in the United States. The bacterium was initially described as a *Rickettsia* due to cross-reactivity with *Cowdria ruminantium* antisera. However, the organism, named WSU 86-1044 was shown to possess a developmental cycle similar to that of the chlamydiae and replicated within intracellular vacuoles [3]. Subsequent phylogenetic analysis of the 16 S gene placed it within the Chlamydiales, and it was named *Waddlia chondrophila* [4]. A causative role in bovine abortion is suggested by its isolation from bovine abortion material in the United States [2] and Germany [5] and through experimental evidence in which administration of the pathogen resulted in bovine fetal death within two weeks [6]. Additionally, serological studies have demonstrated an association between *Waddlia* antibody titres and pregnancy failure in cattle [7] and, more recently, *W. chondrophila* DNA has been identified in vaginal swabs of aborted cattle [8]. Recent evidence has also suggested that this emerging pathogen appears to have a zoonotic potential, with significant implications for human health. A serological study, conducted on women that had experienced sporadic or recurrent miscarriage, demonstrated a strong association between *W. chondrophila* seropositivity and adverse pregnancy outcomes [9]. More recently *W. chondrophila* has been confirmed in the placentas of miscarried human pregnancies by both molecular and immunohistochemical methodologies [10].

In recent *in vitro* studies, *W. chondrophila* has demonstrated the ability to infect a number of cell lines derived from a variety of lineages [11], [12] and also primary human macrophages [13]. Trophoblasts are specialized cells of the placenta that play an important role in embryo implantation and interaction with the maternal uterine tissues. These cells present at the materno-fetal interface play a pivotal role in protecting the fetus from maternally derived pathogens, and their innate immune responses to infection play a significant role towards a successful pregnancy outcome [14]. We have previously demonstrated that the AH-1 ruminant trophoblast cell line responds to *C. abortus* infection through an increase in the secretion of the pro-inflammatory cytokine TNF-α *in vivo* [15]. This current study was carried out to investigate the growth characteristics of *W. chondrophila* in a ruminant trophoblast cell line, and also the innate immune responses of the AH-1 trophoblasts to infection with *W. chondrophila*.

## Materials and Methods

### Cell culture

McCoy cells were obtained from the European Collection of Cell Cultures (ECACC, Salisbury, UK) and maintained in RPMI 1640 medium. The AH-1 cell line was developed by SV40 large T antigen transformation of cells derived from the fetal cotyledon of an ovine placentome, as previously described [16], and was routinely grown in Iscove's Modified Dulbecco's Medium (IMDM, Life Technologies, Paisley, UK). Growth media for both cell lines were supplemented with 5% heat inactivated fetal calf serum (PAA Laboratories Ltd, Yeovil, Somerset, UK).

### Propagation of *W. chondrophila*

*Waddlia chondrophila* strain ATCC VR-1470 was grown at 37°C in McCoy cells with RPMI-1640 medium supplemented with 2% heat inactivated fetal calf serum (PAA Laboratories Ltd,). After 72 hours the cell monolayers were disrupted with glass beads and the medium containing cell debris was removed, before centrifugation at 50×g for 5 minutes at 4°C to remove intact cells. The supernatant was removed and centrifuged at 20,000×g using a J-LITE JLA-16.250 rotor (Beckman Coulter Ltd. High Wycombe, UK). The pellet was resuspended in ice-cold sucrose-phosphate-glutamic acid (SPG) buffer (10 mM sodium phosphate [8 mM Na_2_HPO_4_-2 mM NaH_2_PO_4_], 220 mM sucrose, 0.5 mM l-glutamic acid pH7.4), aliquoted into microcentrifuge tubes and stored at −80°C. To quantify viable organisms, aliquots were thawed at room temperature and titrated on McCoy cells. Serial dilutions of the inoculum were added to confluent cell monolayers in 8-well chamber slides (BD Falcon, Becton Dickinson, Bedford, UK). After 24 hours the medium was removed, cells were fixed in acetone, air-dried, and the slides were frozen at −20°C prior to analysis by fluorescent immunocytochemistry.

### Growth studies

For initial studies examining the infectivity of *W. chondrophila* upon AH-1 cells, the cells were seeded in 8-well chamber slides, and infected at an estimated multiplicity of infection (MOI) of 0.1, 1 and 10. After incubation for 2 hours, the inoculum was removed, and replaced with fresh medium. After a further 24 hours, the medium was removed, and the cells fixed in acetone prior to undergoing fluorescent immunocytochemistry for visualisation of the organisms. For subsequent more detailed growth studies, AH-1 cells were seeded onto 8-well chamber slides for fluorescent microscopy, 24 well plates for DNA extraction (Corning Costar, High Wycombe, United Kingdom), or Thermanox coverslips (Thermo Scientific, Cramlington, UK) for transmission electron microscopy. A single level of infection (MOI 1) was used, and cells were processed at 0, 6, 12, 18, 24, 36, 48 or 60 h post-infection (p.i.).

To determine the timing and extent of cell death after *W. chondrophila* infection, AH-1 cells were grown to approximately 80% confluence in 96 well plates (Corning Costar). The cells were then infected with *W. chondrophila* at an estimated MOI of 0.1, 1 and 10, or exposed to UV-irradiated organisms (MOI 10 equivalent), or uninfected McCoy cell lysate in IMDM containing 2% FCS. At various time-points (12, 24, 36, 48 and 60 h post-infection), plates were processed to determine levels of lactate dehydrogenase (LDH) release using a Pierce LDH Cytotoxicity Assay Kit (Thermo Scientific) as a measure of cell death.

### Fluorescent Immunocytochemistry

To visualise the organisms, slides were removed from −20°C storage and rehydrated in PBS for 5 min before blocking in a 2% BSA in PBS solution for 30 min at RT. The slides were incubated for a further 60 min with rabbit anti-*W. chondrophila* serum at RT. After washing three times in PBS, the chambers were removed and the slides were incubated with a goat anti-rabbit FITC conjugated antibody (Sigma Aldrich, Poole, UK) for 30 min at RT in a light-tight humidity chamber, before washing a further three times in PBS and mounting using Prolong Gold anti-fade reagent containing DAPI (Life Technologies). During initial experiments slides were incubated with a Phalloidin-Atto 550 conjugate (Sigma) to stain host actin filaments prior to the mounting step. Slides were examined using a digital imaging system with an Axioscope fluorescent microscope (Carl Zeiss Ltd, UK) equipped with GFP and DAPI fluorescent filter sets and Cell* Imaging Software (Soft Imaging Systems, Münster, Germany) for image capture.

### Electron microscopy

AH-1 cells on coverslips (Thermanox) were fixed in 2.5% glutaraldehyde in 0.1 M sodium cacodylate (pH7.4) for 2 hours at 4°C. After fixation the coverslips were washed twice in ice-cold PBS. Specimens were post-fixed in 1% osmium tetroxide in 0.1 M sodium cacodylate for 45 minutes, before washing three times in 0.1 M sodium cacodylate buffer (pH7.4). Samples were dehydrated in 50%, 70%, 90% and 100% normal grade acetone solutions for 10 minutes each, before two further 10-minute changes in analar grade acetone. Samples were then embedded in Araldite resin. 1 µm thick sections were cut on a Reichert OMU4 ultramicrotome, stained with toluidine blue, and viewed in a light microscope to select representative areas for investigation. Ultrathin sections of 60 nm thick were cut from selected areas, stained in uranyl acetate and lead litrate and viewed in a Philips CM120 transmission electron microscope (Philips Research, Cambridge, UK). Images were captured on a Gatan Orius CCD camera (Gatan UK, Oxon, UK).

### Quantification of *W. chondrophila* growth

DNA was isolated using the DNeasy Blood and Tissue kit (Qiagen, Crawley, UK). At each time-point the medium was removed from each well and centrifuged at 14,000 rpm for 5 minutes in a microfuge. The monolayers were lysed directly in 200 µl AL buffer (supplied with DNeasy Blood and Tissue kit). The cell lysate and pellet resulting from centrifugation of the medium were combined and thoroughly mixed. The combined lysate was mixed sequentially with 200 µl PBS and 20 µl Proteinase K before incubation at 55°C for 10 minutes. 200 µl absolute ethanol was added to each sample and DNA extracted according to the manufacturer's instructions.

A pan-Chlamydiales qPCR targeting the 16 S rRNA gene was performed to quantify the replication of the organism in culture. The qPCR assays were performed as previously described [17] using the forward primer panCh16F2 (5′-CCGCCAACACTGGGACT-3′), the reverse primer panCh16R2 (5′-GGAGTTAGCCGGTGCTTCTTTAC-3′) and the probe panCh16S (5′-FAM-CTACGGGAGGCTGCAGTCGAGAATC-BHQ1-3′). Assays were performed in a total volume of 20 µl, using the Quanta Toughmix Low ROX (Quanta BioSciences, Inc., Gaithersburg, USA), 0.1 µM primer (Exiqon, Vedbaek, Denmark), a 0.1 µM probe (Integrated DNA Technologies, Iowa, USA), molecular-biology-grade water (Promega, Southampton, UK), and 1 µl DNA. The cycling conditions were 3 min at 95°C, followed by 50 cycles of 15 s at 95°C, 15 s at 67°C and 15 s at 72°C. The PCR products were detected with an ABI 7500 (Life Technologies). Molecular grade water was used as a negative PCR control.

Quantification was achieved using a standard curve derived using a recombinant plasmid control as previously described [17]. DNA from *Parachlamydia acanthamoebae* strain Bn9 (ATCC) was isolated from a purified bacterial culture, using a DNeasy Blood and Tissue Kit (Qiagen). A PCR was performed using AmpliTaq Gold Mastermix (Life Technologies, Paisley, UK) and the primers Pacstd16SF2 (5′-GCTGACGGCGTGGATGAGGC-3′) and Pacstd16SR2 (5′-CCTACGCGCCCTTTACGCCC-3′) as previously described [17]. The PCR products were gel purified using an ISOLATE PCR and Gel Kit (Bioline Ltd, London, UK) and cloned into pGEM-T (Promega, Southampton, UK). Plasmids were transformed into Escherichia coli JM109 competent cells (Promega) and successfully transformed cells were selected by ampicillin resistance and blue-white colony selection, according to standard procedures. Positive colonies were grown in overnight broths. Isolation of plasmid DNA was performed using an ISOLATE Plasmid Mini Kit (Bioline, London, UK). The insertion of PCR products was demonstrated by restriction digestion, prior to confirmation by sequencing. Sequencing was performed using primers directed against the T7-promoter sequence of the plasmid by dideoxy chain termination / cycle sequencing (Eurofins MWG Operon, Ebersberg, Germany). Quantification of the recombinant plasmid was made on a Nanodrop ND-1000 (ThermoFisher Scientific, Leicestershire, UK) and 10-fold dilutions (10^7^ copies to 1 copy/µl) were used to establish a standard curve for quantification. Negative controls, the standard curve, and samples were all analyzed in triplicate.

### Cytokine expression analysis

To investigate the effect of *W. chondrophila* on cytokine expression by ovine trophoblasts, cells were grown to approximately 80% confluence in 24 well plates. The cells were then infected with *W. chondrophila* at an estimated MOI of 0.1, 1 and 10 or exposed to UV-killed organisms (MOI 10 equivalent) or an uninfected cell lysate in IMDM containing 2% FCS. The inoculum was removed 2 h post infection, and was replaced with fresh medium. At 24 h p.i. the medium was removed and the cells were lysed in RLT plus buffer for subsequent DNA and RNA isolation using the RNeasy Plus miniprep kit (Qiagen).

The isolated RNA concentration was determined using a Nanodrop spectrophotometer (ThermoFisher Scientific) and integrity determined using an Agilent 2100 Bioanalyzer (Agilent Technologies, Edinburgh, UK). To remove residual contaminating DNA, the RNA was treated using a Precision DNase kit (PrimerDesign, Southampton, UK). 1 ug of total RNA was reverse transcribed using the Precision nanoScript reverse transcription kit. Gene expression was analysed using the Precision 2×qMastermix with SYBR Green (PrimerDesign) and run on an Applied Biosystems StepOne Plus Real-Time PCR platform (Applied Biosystems) for 10 mins at 95°C, the 40 cycles of 15secs at 95°C, and 1 min at 60°C. Forward and reverse primers were used at a final concentration of 900 nM. Sequences for TNF-α, CXCL8 and IL-1β primers [18] and IL-1α [19] have been previously published. Samples were analyzed in triplicate using 50 ng cDNA. Melting curve analysis revealed a single amplicon in all cases. GeNorm analysis of 12 ovine reference genes (PrimerDesign Ltd, UK) revealed that two housekeeping genes were required to give robust housekeeping stability; hence the geometric mean of GAPDH and ribosomal 18 S was used as the normalization reference. Gene expression was quantified using the 2^−(ΔΔCt)^ method.

### Statistics

Data were log transformed and analysed by two-way ANOVA. Comparisons between individual treatments were made using Tukey's multiple comparison test. All analyses were performed using GraphPad Prism 6 (GraphPad Software, Inc. La Jolla, California, USA).

## Results

### Fluorescent Immunocytochemistry

**Figure 1:**
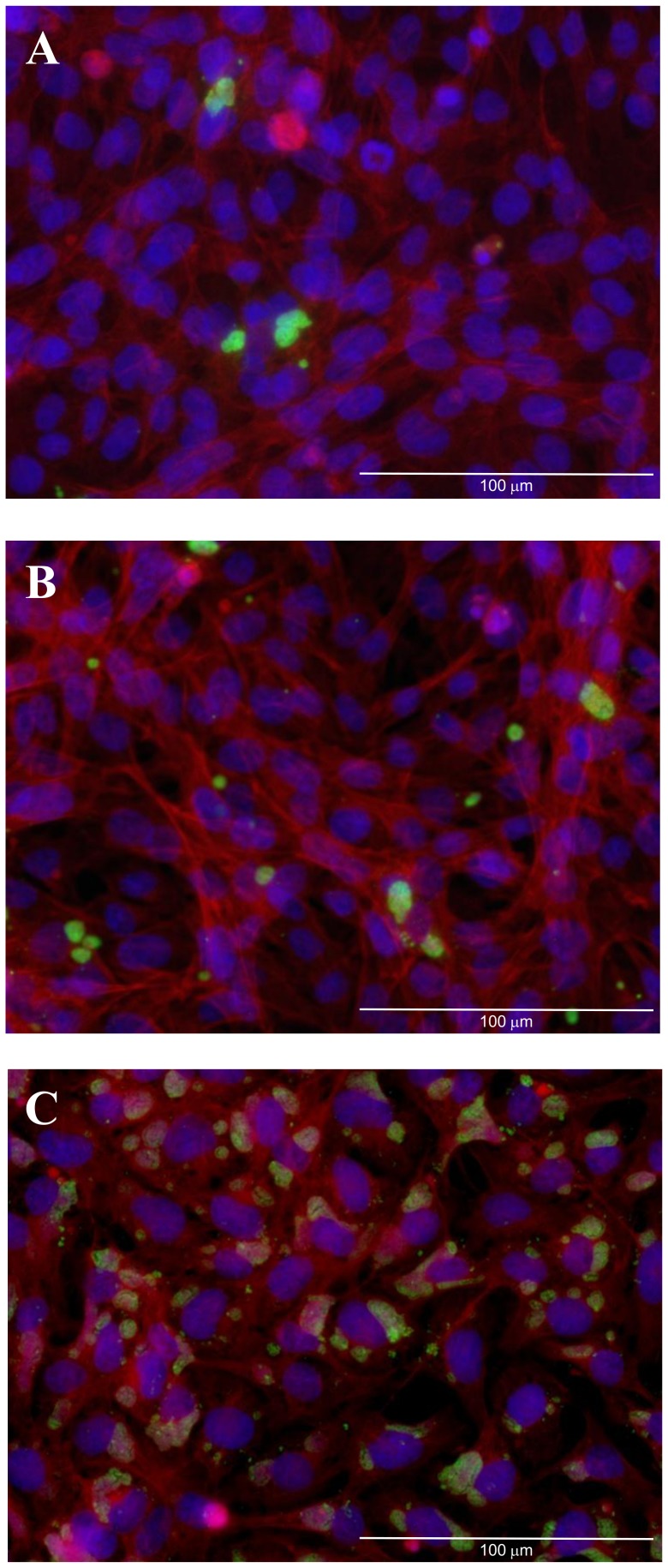
Fluorescent micrographs demonstrating the infectivity of *W. chondrophila* at an apparent MOI of 0.1 (A), 1 (B) and 10 (C) at 24 h post-infection. *W. chondrophila* inclusions are labelled green using an anti-*Waddlia* rabbit polyclonal antisera and FITC anti-rabbit secondary antibody, host cell nuclei are stained in blue (Dapi) and host actin-filaments labelled with Phalloidin-Atto 550 (red). The scale bars correspond to 100 µm.

**Figure 2:**
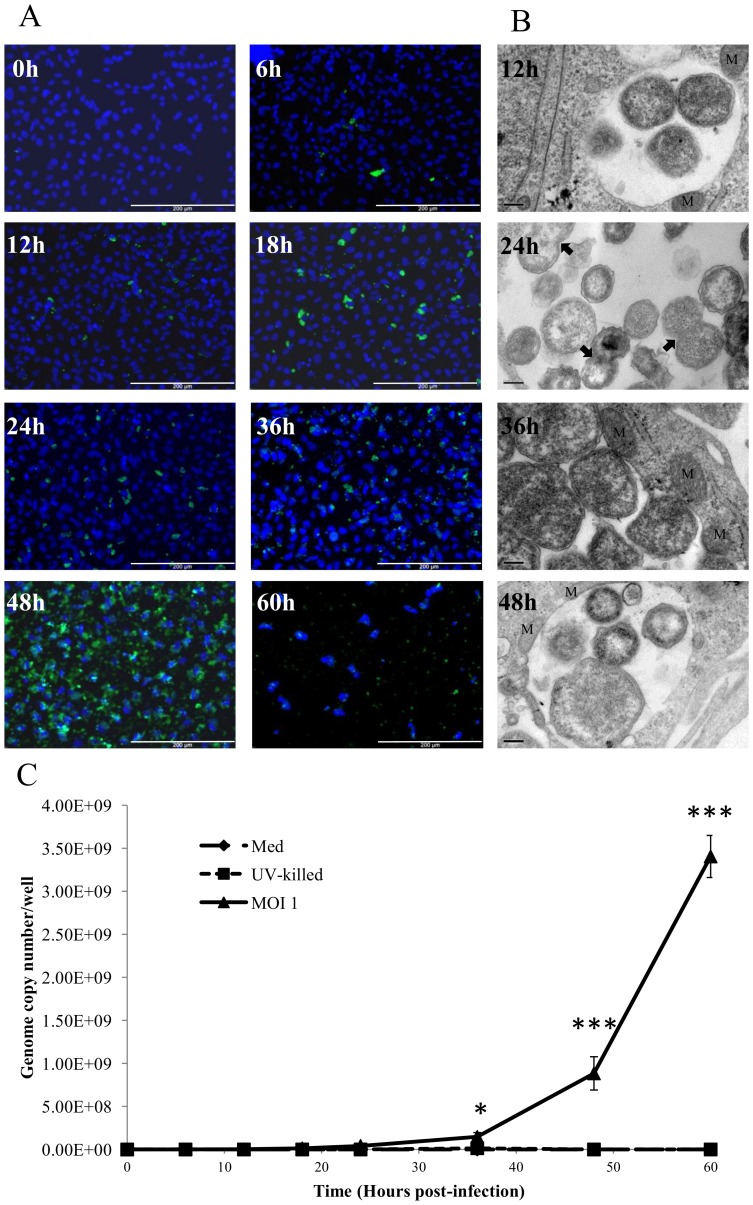
Bacterial growth within AH-1 trophoblast cells. A) Growth of *W. chondrophila* in AH-1 cells assessed by fluorescence microscopy. *W. chondrophila* inclusions are labelled green using an anti-*Waddlia* rabbit polyclonal antisera and FITC anti-rabbit secondary antibody, host cell nuclei are stained blue (Dapi). The scale bars correspond to 200 µm. B) Transmission electron micrographs at various specified time-points post-infection, demonstrating RB dividing by binary fission (24 h panel indicated by the arrows) and mitochondria associated with the inclusion (12 h, 36 h and 48 h panels indicated by M) and multiple forms of the organism (48 h panel) as the RBs condense to EBs. C) Quantitation of *W. chondrophila* growth by qPCR for 60 h post-infection. AH-1 cells were exposed to live *W. chondrophila* (MOI 1), UV- killed organisms (UV-killed) or medium alone (Med) and DNA isolated at the indicated time-points over the 60 h time-course. Statistically-significant differences relative to initial genome copy numbers at 0 h post-infection are indicated by *P<0.05; ***P<0.001 Data were analysed by two-way ANOVA. Comparisons between individual treatments were made using Tukey's multiple comparison test (n = 3).

Initial experiments indicated that the relative infectivity of *W. chondrophila* was 3.6%, 36% and 88% at MOI's 0.1, 1 and 10 respectively 24 hours p.i. (Figure 1). A time-course of *W. chondrophila* infectivity using an MOI 1 revealed the presence of a small number of visible inclusions as early as 6 h p.i.. This was followed by a gradual increase in the number of visible inclusions in the cell monolayer up to 48 h p.i. (Figure 2A). At 60 h p.i. the number of stained host cell nuclei and level of visualised *W. chondrophila* antigen were reduced indicating lysis of the host cells.

### Electron Microscopy

Transmission electron microscopy was used to visualise the organisms throughout the developmental cycle. The presence of RBs could be visualised by 12 h p.i. (Figure 2B). The inclusions appeared to gradually increase in size throughout the cycle with increased numbers of RBs indicating replication of the organisms. The inclusion membrane remained closely associated with the host mitochondria, as previously described [11]. At 48 h p.i. multiple forms of the organism were visible inside the inclusion consistent with the condensation of RBs to EBs (Figure 2B). At 60 h p.i. no intact infected cells could be visualised on the coverslip.

### Quantification of the replication of *W. chondrophila*

The replication of the organism was quantified by qPCR of total DNA targeting the *W. chondrophila* 16 s rRNA gene. Growth of the organism appeared to occur in an exponential manner (Figure 2C). When compared to the initial 0 h p.i. time point an increase in genome copy number was observed at all time points, however this was not significant until 36 h p.i. (2.13×10^5^ at 0 h vs 1.47×10^8^ at 36 h, P<0.05) attaining a greater than 4 log increase by 60 h p.i. (2.13×10^5^ vs 3.41×10^9^, P<0.001). As expected there was no observed increase in genome copy number of UV-killed organisms at any of the time points.

### Host cell death (LDH release)

**Figure 3:**
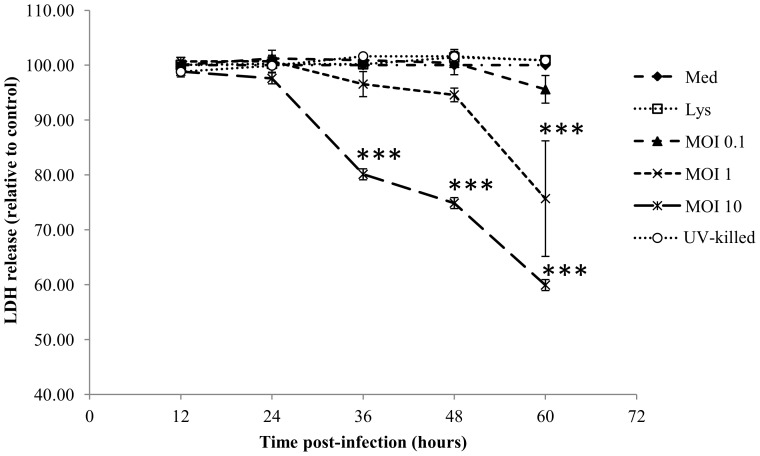
Changes in host cell viability over time after exposure of AH-1 trophoblasts to medium alone (Med), uninfected control cell lysate (Lys) or increasing MOI of *W. chondrophila* (MOI 0.1, 1, 10) or UV-killed organisms (MOI 10) quantified by lactate dehydrogenase release (n = 3). Statistically-significant differences relative to medium control at each time-point are indicated by ***P<0.001. Data were analysed by two-way ANOVA. Comparisons between individual treatments were made using Tukey's multiple comparison test.

Release of LDH from cells was quantified over the 60 h period post-infection (Figure 3). There was no significant detection of cell death with exposure of host cells to the control lysate, UV-killed *W. chondrophila* or those infected with an MOI 0.1. At an MOI 10 (initial infection rate of approximately 88%), significant levels of LDH release could be observed at all time points from 36 h pi onwards (P<0.001), indicating that host cell lysis did not occur prior to this time-point. At 60 h p.i. infection with either an MOI 1 or MOI 10 significantly increased quantifiable LDH release of host cells compared to controls by 34%, and 70% respectively (P<0.001) (Figure 3).

### Cytokine expression

**Figure 4:**
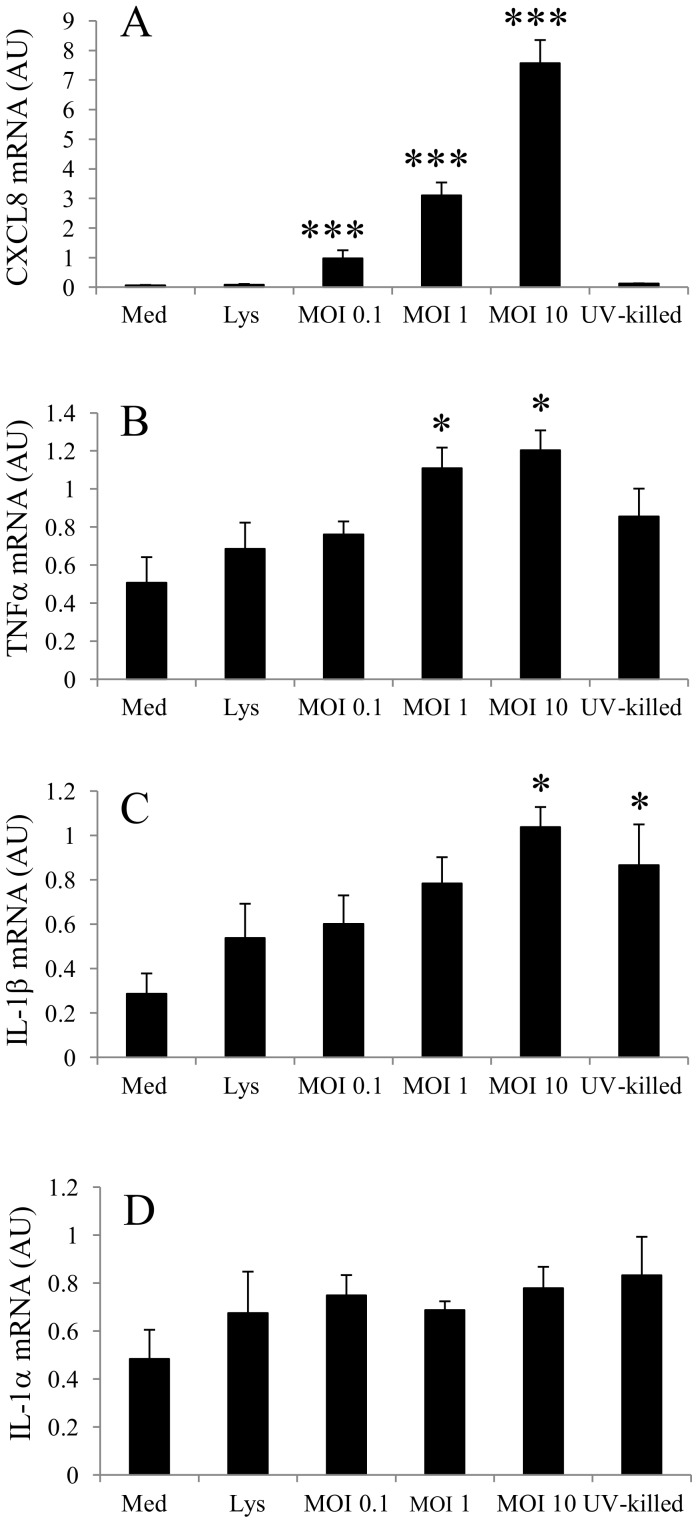
Changes in the expression of A) CXCL8, B) TNF-α, C) IL-1β, D) IL-1α mRNA after exposure of AH-1 trophoblast cells to medium alone (Med), uninfected control cell lysate (Lys) or increasing MOI of *W. chondrophila* (MOI 0.1, 1, 10) or UV-killed organisms (MOI 10) for 24 h. Statistically-significant differences relative to medium control are indicated by *P<0.05; ***P<0.001. Data were analysed by two-way ANOVA. Comparisons between individual treatments were made using Tukey's multiple comparison test (n = 3). Data are presented as arbitrary units (AU).

Infection of trophoblasts with chlamydial species initiates a pro-inflammatory response, which is thought to be at least partially responsible for the pathogenic effects of the organism *in vivo*. Therefore, the effects of *W. chondrophila* infection upon the expression of the pro-inflammatory cytokines, TNF-α, IL-1α, IL-1β and the chemokine CXCL8 was investigated 24 h post-infection. CXCL8 expression was significantly increased by exposure of AH-1 cells to the organism in a dose dependent manner (P<0.001) (Figure 4A) when compared to the negative control. However, exposure of the cells to either a control lysate or UV-killed organisms (MOI 10) failed to elicit a response in CXCL8 expression.

A pattern of increased TNF-α expression was observed to be statistically significant at an MOI 1 and 10 (P<0.05) (Figure 4B). Similarly to CXCL8 expression, UV-killed organisms failed to elicit a significant response in terms of increased TNF-α expression. Notably, IL-1β expression was significantly elevated by *W. chondrophila* infection at an MOI of 10 (P<0.05); although UV-treated organisms at the same MOI also significantly increased expression to a comparable level compared to controls (P<0.05) (Figure 4C). Conversely, and compared to the other effects of *W. chondrophila* infection on expression of the other cytokines, there was no significant response in terms of IL-1α expression to any of the treatments (Figure 4D).

## Discussion

*Waddlia chondrophila* is considered a potential zoonotic abortifacient agent and has now been identified in the placentas of aborted foetuses in both cattle [2] and humans [10]. The current study utilised a ruminant placental cell line to investigate the growth of the organism within placental trophoblast cells and investigate the innate immune responses to infection.

The AH-1 cell line was found to be highly permissive to infection with the organism, and once infected, it was demonstrated that *W. chondrophila* undergoes a classical chlamydial developmental cycle within these cells. The rapid conversion of elementary bodies to metabolically active reticulate bodies was clearly visible by 12 h post-infection. However, with a small number of inclusions visible by standard fluorescent immunocytochemistry within 6 h of infection, it is likely that this conversion from EB to RB could occur earlier in the cycle than observed for classical chlamydiae. An exponential increase in genome copy number was observed throughout the cycle and almost immediately following infection, which is consistent with previous studies in human cell lines [11]. This increase in genome number was accompanied by a concomitant decrease in cell viability, and with a decrease in the number of attached cells visible after immunocytochemistry at 60 h p.i.. This observation was quantified by the release of cytosolic lactate dehydrogenase into the culture medium between 36-60 h p.i. indicating host cell lysis and release of EBs.

Electron microscopy of infected cells at different time-points in the developmental cycle of the organism demonstrated the intimate association of *W. chondrophila* inclusions with host cell mitochondria as previously observed in human cell lines [11] and macrophages [20]. Intracellular bacteria have developed sophisticated mechanisms to interact and exploit their hosts, such as the expression of transport proteins that enable the acquisition and utilisation of host cell resources. *W. chondrophila* appears to possess an enhanced ability to generate ATP independently from its host compared to other members of the Chlamydiales through encoding genes for a complete TCA cycle, and capability to utilise oxidative phosphorylation pathways. However, as with many other intracellular pathogens, including members of the ricketsiae and the chlamydiae [21] the genome encodes an ATP/ADP translocase, which enables the organism to import host ATP in exchange for bacterial ADP [22]. It has been suggested that being in such close proximity to the mitochondria, and rich source of ATP, may confer an advantage to the organism [11].

The innate immune response to infection of the AH-1 cells to infection by *W. chondrophila* is similar to that observed with *C. abortus* [15]. Similarly, the mechanisms by which AH-1 cells recognise *W. chondrophila infection* and induce the expression of CXCL8 and TNF-α appear to be dependent on active infection and intracellular invasion rather than recognition by cell-surface expressed pattern recognition receptors, as UV-killed organisms failed to elicit the same effect as live organisms. This is consistent with observations on human ectocervical cells that secrete CXCL8 in response to live but not UV-killed *C. trachomatis* serovar L2, with the response being attributed to the intracellular association of TLR2 with the chlamydial inclusion [23]. In addition to TLR2, the NOD1 intracellular pattern recognition receptor, which detects specific motifs in bacterial peptidoglycan, has also been implicated in the induction of a CXCL8 response to chlamydial infection [24], [25]. Our previous work on *C. abortus* infection of AH-1 cells measured protein secretion rather than mRNA expression, and thus it is difficult to make direct comparisons in the context of this study. However, it is notable that significant induction of expression of CXCL8 occurs much more rapidly after *W. chondrophila* infection (24 h) than after *C. abortus* infection (72 h). This is may be due to differences in the lengths of developmental cycle of the two organisms, which appears to be substantially shorter for *W. chondrophila* (36–60 h) than *C. abortus* (72–96 h). The timing of CXCL8 expression after *W. chondrophila* infection appears to be closer to that of *C. trachomatis* [26], which also exhibits a relatively rapid developmental cycle.

Conversely, induction of IL-1β expression appeared to be of a similar magnitude after exposure to either live or UV- killed bacteria, indicating potential surface recognition of the organism. This is in contrast to recent evidence that suggested IL-1β expression and secretion after infection of human trophoblasts with *C. trachomatis* was dependent upon intracellular NOD1 [27]. The potential differences in the innate immune response to infection may either be due to the use of cells from different host species or perhaps because of fundamental differences between the pathogens. *W. chondrophila* is a member of the Chlamydiales and its morphology and developmental cycle shares many similarities with members of the Chlamydiaceae. However, the organism does differ in many respects from these related species at a genomic [22] and proteomic [28] level. These differences include an extended family of OmpA proteins and a lack of Pmp proteins. However, the genome also encodes additional enzymes for cell wall biosynthesis and it has been speculated that this may influence membrane structure and host cell recognition [22].

This study demonstrates that *W. chondrophila* infects and multiplies in ruminant trophoblast cells. Furthermore, this infection actively stimulates an innate immune response which results in increases in expression of CXCL8 and TNF-α that have been implicated in ovine abortion following *C. abortus* infection [15]. It addition, we have demonstrated that exposure of the cells to UV-killed organisms stimulates expression of pro-inflammatory IL-1β , indicating the stimulation of several innate immune pathways by the organism. Further *in vitro* and *in vivo* investigations are required to understand the mechanisms through which *W. chondrophila* induces expression of pro-inflammatory molecules, which will aid our understanding of the immunopathogenic mechanisms that lead to abortion.
